# First report of the genus *Conostigmus* Dahlbom (Hymenoptera: Ceraphronoidea: Megaspilidae) from India with description of a new species

**DOI:** 10.3897/BDJ.2.e991

**Published:** 2014-01-02

**Authors:** C Bijoy, K Rajmohana, Ramesh Kumar

**Affiliations:** † Western Ghats Regional Centre, Zoological Survey of India, Calicut-673006, Kerala, India; ‡ National Bureau of Agriculturally Important Insects, Hebbal, Bangalore-560024, Karnataka, India

**Keywords:** *
Conostigmus
*, Megaspilidae, new species, India

## Abstract

The genus *Conostigmus* Dahlbom (Hymenoptera: Ceraphronoidea: Megaspilidae) is reported for the first time from India, along with description and illustration of *Conostigmus neotubifer* sp. n. A comparative discussion on the morphological affinities of the new species with its putative sister *Conostigmus tubifer* Dessart is provided. An intermixing of character states of genera *Conostigmus* as well as *Dendrocerus* Ratzeburg is observed.

## Introduction

Superfamily Ceraphronoidea (Hymenoptera) comprises two extant families of parasitoid wasps, Ceraphronidae and Megaspilidae (Johnson and Musetti 2004). Megaspilidae includes 305 valid species in 13 genera (http://hol.osu.edu/index.html?id=91). The species of Megaspilidae are generally ectoparasitoids attacking cocoons and puparia, primarily on Diptera, Neuroptera, Coleoptera, Mecoptera and also as hyperparasitoids of Hymenoptera (Dessart 1995).

Among the 13 genera of Megaspilidae, the most speciose and widely distributed are *Dendrocerus* Ratzeburg 1852 and *Conostigmus* Dahlbom 1858. The genus *Conostigmus* was erected by Dahlbom (1858), as a subgenus of *Megaspilus* Westwood, with *Megaspilus alutaceus* Thomson as type species. Later in 1951, Muesebeck and Walkley elevated the taxon to generic level. The genus is distributed widely in all geographical realms and has 168 species. Only 9 species have been reported from Oriental region so far (Johnson and Musetti 2004, Dessart 1997) (catalogued as 8 species by Johnson and Musetti (2004), missing out *Conostigmus occipitalis* Dessart 1997).

In continuation with our pioneer taxonomic studies on the superfamily Ceraphronoidea of India, a new species of *Conostigmus* viz. *Conostigmus neotubifer* sp. n. is hereby described and illustrated. A comparative discussion on the morphological affinities of the new species with *Conostigmus tubifer* Dessart 1997, the most resembling species is also provided. Both the species are remarkable for their intermixing of characters seen in two genera, *Conostigmus* and *Dendrocerus*.

## Materials and methods

The specimens under study were collected using malaise trap set among the grassy patches of Port Blair, Andaman and Nicobar Islands, India. The specimens were card mounted on point-card tips. Description and imaging works were carried out employing Leica M205A stereomicroscope, with 1 × objective and Leica DFC-500 digital camera. Morphological terminology follows Fergusson (1980), Dessart (1997) and Miko and Deans (2009). Based on Dessart (1997), we have attempted to define the various states of ocellar triangle in Megaspilidae. If OOL > POL, the ocellar triangle is ‘narrow-based’ and if OOL < POL, the triangle is ‘broad-based’. If POL = LOL, then the ocellar triangle is ‘Equilateral’, and if POL is not equal to LOL, the triangle is ‘Isosceles’, the condition most common in occurrence. The triangle is said to be ‘short’ when LOL < POL and ‘long’ when LOL > POL.

Both the Holotype and the Paratypes are deposited in the National Zoological Collection at Zoological Survey of India, Calicut.

**Abbreviations:** DFIm – Minimal Interocular Facial Distance; OOL – Ocellocular Length; POL – Postocellar Length; LOL – Lateral Ocellar Length; T3 – Metasomal tergite 3.

NHM – Natural History Museum, London.

## Taxon treatments

### Conostigmus neotubifer

Bijoy & Rajmohana, 2014 sp. n.

urn:lsid:zoobank.org:act:4BF76167-8B66-4FB5-B4C1-DEB4C9FBF302

#### Materials

- **Type status:** Holotype. **Occurrence:** recordedBy: Ramesh Kumar; individualID: ZSI/WGRC/IR/INV.2652; individualCount: 1; sex: male; lifeStage: adult; preparations: Card mount; **Taxon:** scientificNameID: Conostigmus neotubifer; kingdom: Animalia; phylum: Arthropoda; class: Insecta; order: Hymenoptera; family: Megaspilidae; genus: Conostigmus; specificEpithet: neotubifer; scientificNameAuthorship: Bijoy and Rajmohana; **Location:** continent: Asia; island: Andaman and Nicobar island; country: India; stateProvince: Andaman and Nicobar island; locality: Port Blair; verbatimLocality: Bathu Barthi; decimalLatitude: 11.768 N; decimalLongitude: 92.737 E; **Identification:** identifiedBy: Bijoy and Rajmohana; dateIdentified: 06/27/2013; **Event:** habitat: grassy field; eventRemarks: collected in malasie trap; **Record Level:** institutionID: ZSIC
- **Type status:** Paratype. **Occurrence:** recordedBy: Ramesh Kumar; individualID: ZSI/WGRC/IR/INV.2653; individualCount: 1; sex: female; lifeStage: adult; preparations: Card mount; **Taxon:** scientificNameID: Conostigmus neotubifer; kingdom: Animalia; phylum: Arthropoda; class: Insecta; order: Hymenoptera; genus: Conostigmus; specificEpithet: neotubifer; scientificNameAuthorship: Bijoy and Rajmohana; **Location:** continent: Asia; island: Andaman and Nicobar island; country: India; stateProvince: Andaman and Nicobar island; locality: Port Blair; verbatimLocality: Bathu Barthi; decimalLatitude: 11.768 N; decimalLongitude: 92.737 E; **Identification:** identifiedBy: Bijoy and Rajmohana; dateIdentified: 06/27/2013; **Event:** habitat: grassy field; eventRemarks: collected in malasie trap; **Record Level:** institutionID: ZSIC
- **Type status:** Paratype. **Occurrence:** recordedBy: Ramesh Kumar; individualID: ZSI/WGRC/IR/INV.2654; individualCount: 1; sex: female; lifeStage: adult; preparations: Card mount; **Taxon:** scientificNameID: Conostigmus neotubifer; kingdom: Animalia; phylum: Arthropoda; class: Insecta; order: Hymenoptera; genus: Conostigmus; specificEpithet: neotubifer; scientificNameAuthorship: Bijoy and Rajmohana; **Location:** continent: Asia; island: Andaman and Nicobar island; country: India; stateProvince: Andaman and Nicobar island; locality: Port Blair; verbatimLocality: Bathu Barthi; decimalLatitude: 11.768 N; decimalLongitude: 92.737 E; **Identification:** identifiedBy: Bijoy and Rajmohana; dateIdentified: 06/27/2013; **Event:** habitat: grassy field; eventRemarks: collected in malasie trap; **Record Level:** institutionID: ZSIC

#### Description

##### Holotype: Male

(Fig. 1).

**Figure 1.**
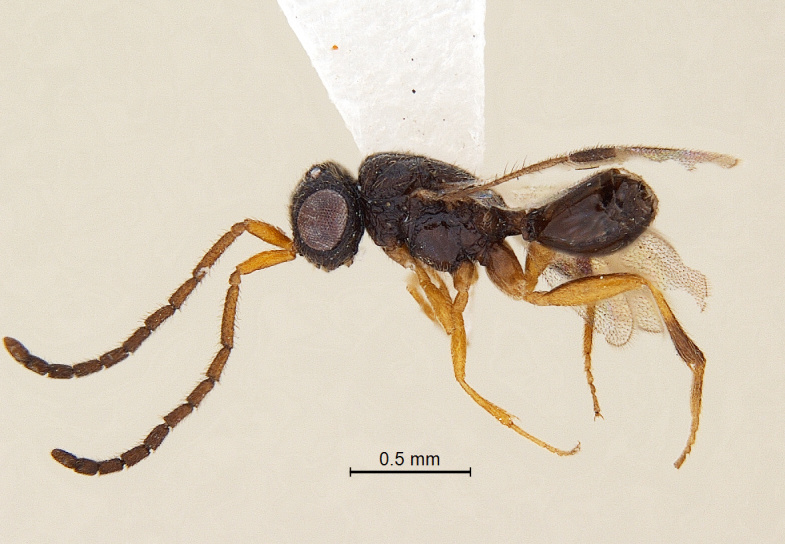
Male habitus, lateral view.

**Coloration**: Body blackish brown with head more darker; eyes and ocelli silvery with a bronze tinge; fore wing clear at base, with large infuscate area below pterostigma and radial vein extending to its posterior margin; scape, pedicel and A3 brownish yellow turning darker towards segments from A4 onwards; legs brownish yellow with coxae and posterior half of hind tibia blackish brown (Fig. 1); mandible light brown with darker distal extreme; pterostigma brown getting darker to wing margin; radial vein and costal vein pale brown (Fig. 8); body pubescence white.

**Body:** length: 1.52 mm.

**Head:** (length/width/height: 230/580/460); eye (length/width: 260/210); preoccipital crescent well-separated from posterior ocelli merging laterally into eye margin (Fig. 3); temple strongly carinated; preoccipital furrow present; ocellar triangle isosceles, narrow, short and raised; LOL < POL; hind ocelli remote from eye margin: POL/LOL/OOL: (80/40/110) Fig. 3. DFIm 57% of head width. Head 1.28 × wider than mesosoma (Fig. 5); facial sulcus present, extending to intertorular carina (Fig. 4); supraclypeal depression absent. Intertorular carina clear with a slightly elevated median peak (Fig. 3); ocular suture prominent and foveolate; eyes densely pubescent (Fig. 4); frons with sparse hairs; clypeus narrow and rectangular with angulated corners (Fig. 7); mandible long, bidendate, lower tooth distinctly longer than upper one.

**Figure 2.**
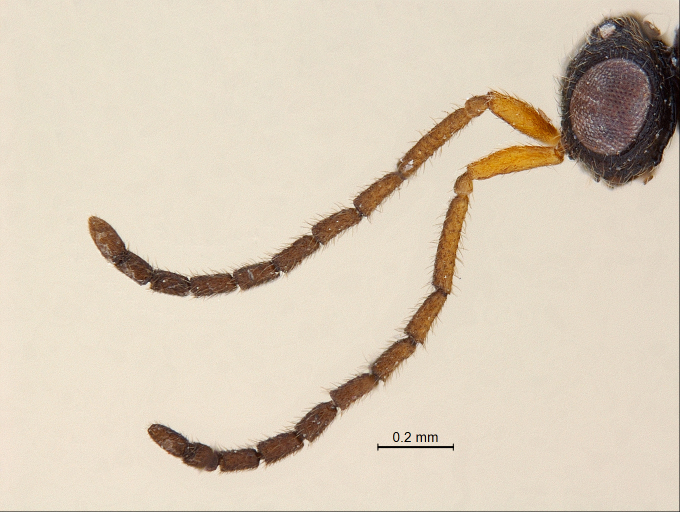
Male antenna, lateral view.

**Figure 3.**
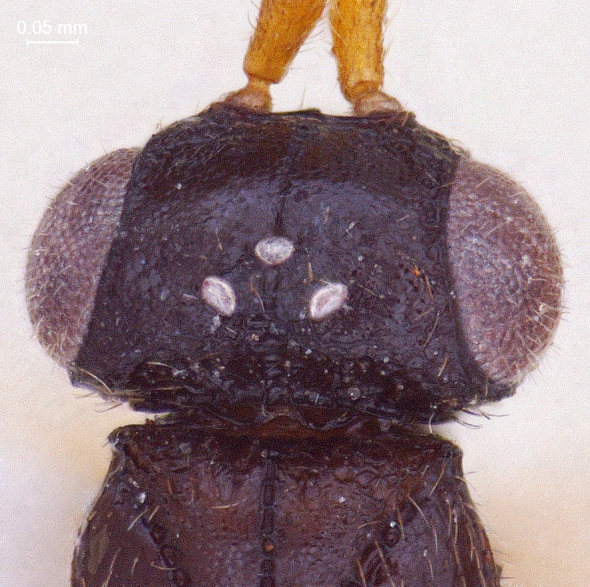
Male head, dorsal view.

**Figure 4.**
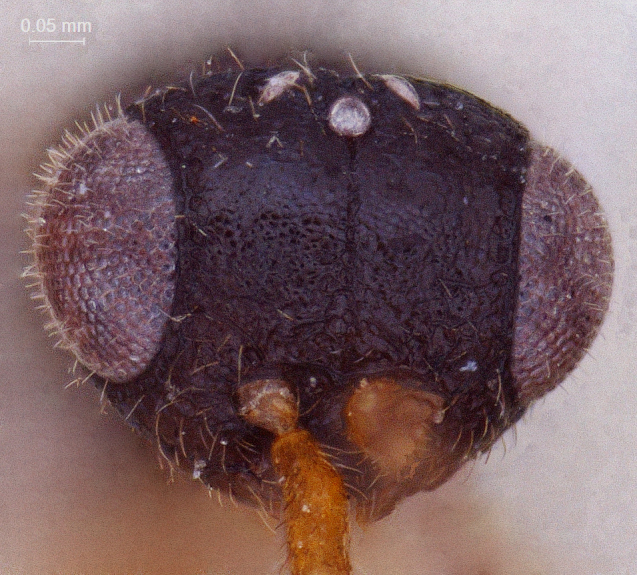
Male head, anterior view.

**Antenna:** (Fig. 2); very long (subequal to the body length) and slender; scape nearly 4 × longer than wide; pedicel small and almost globular; A3 highly slender, nearly 4.8 × longer than wide, subequal to scape; flagellar segments quasi cylindrical with very slight serrations basally; pubescence shorter than breadth of segments; length/width measurements of antennal segments: scape (252/66), pedicel (60/48), A3 (255/53), A4 (147/54), A5 (140/53), A6 (122/53), A7 (128/61), A8 (117/64), A9 (108/57), A10 (82/70), A11 (127/54).

**Figure 5.**
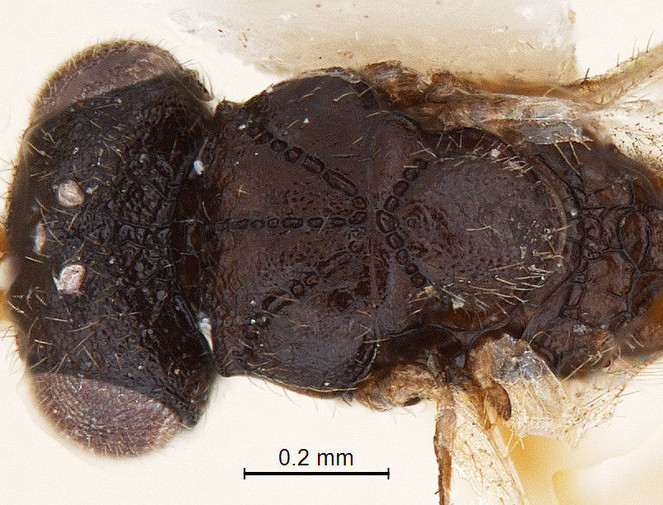
Male head and mesosoma, dorsal view.

**Figure 6.**
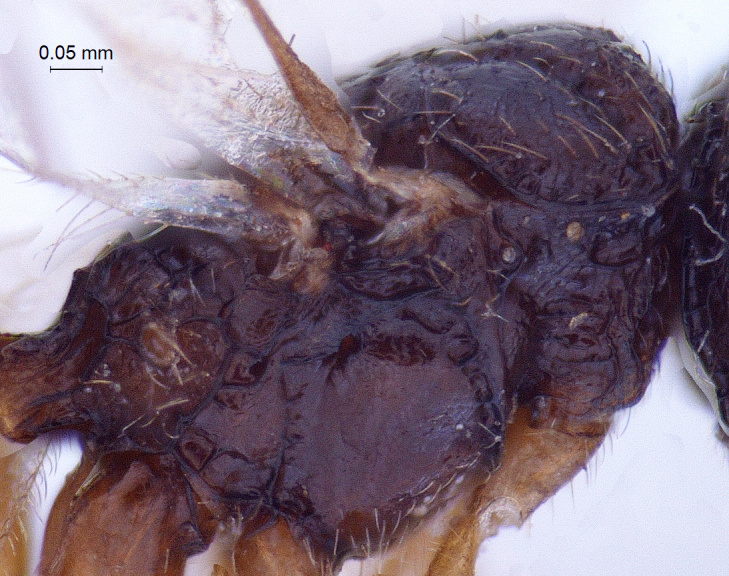
Male mesosoma, lateral view.

**Figure 7.**
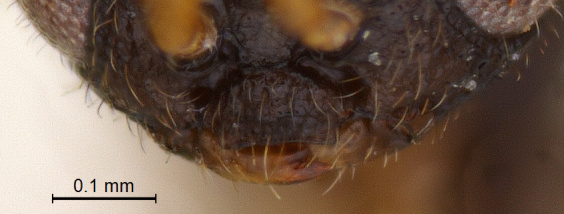
Male clypeus, anterior view.

**Mesosoma:** (length/width/height: 610/450/460); mesosoma robust with very angular shoulders; alutaceous in sculpture; mesoscutum: (length/width: 230/440); mesoscutum nearly 2 × wider than long; lateral margin of mesoscutum posterior to anterior margin of notaulus convex; coarsely foveolate notaulices curve smoothly and converging posteriorly, meeting median furrow at transscutal articulation (Fig. 5); scutellum broadly rounded at apex with long hairs; axilliluar carinae present; metanotal-propodeal sulcus placed in regular intervals medially and with some longitudinal carina remote laterally (Fig. 9); propodeum unarmed, smooth and bare with sculpture effaced and with irregular carinae (Fig. 9) but stretched posteriorly into a inverted ‘U’ shaped narrow apex. Lateral propodeal carinae present. Propodeal spiracle large and conspicuous (Fig. 1); sternaulus absent (Fig. 6); anterior mesopleural sulcus and meso-metapleural sulcus distinct and foveolate; metapleuron bordered by incomplete carina; lower margin of mesopleuron and metapleuron with dense hairs.

**Figure 8.**
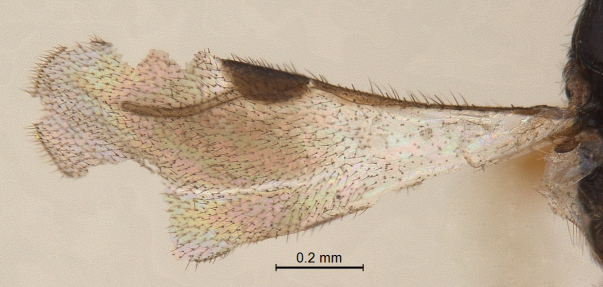
Male forewing.

**Figure 9.**
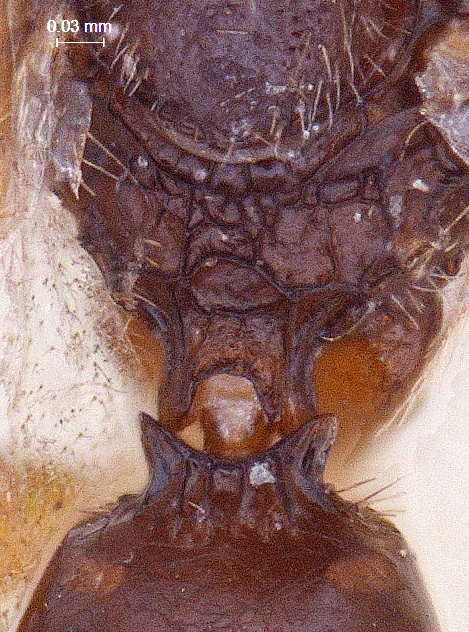
Male propodeum.

**Forewing:** (Fig. 8); total wing length 1.25 mm. Pterostigma (length/width: 200/80) semielliptical, 2.4 × longer than wide. Radius (0.27 mm), slightly curved and 1.36 × the length of pterostigma; maximal width of fused costal and subcostal vein wider than radius; basal part with less hairs.

**Figure 10.**
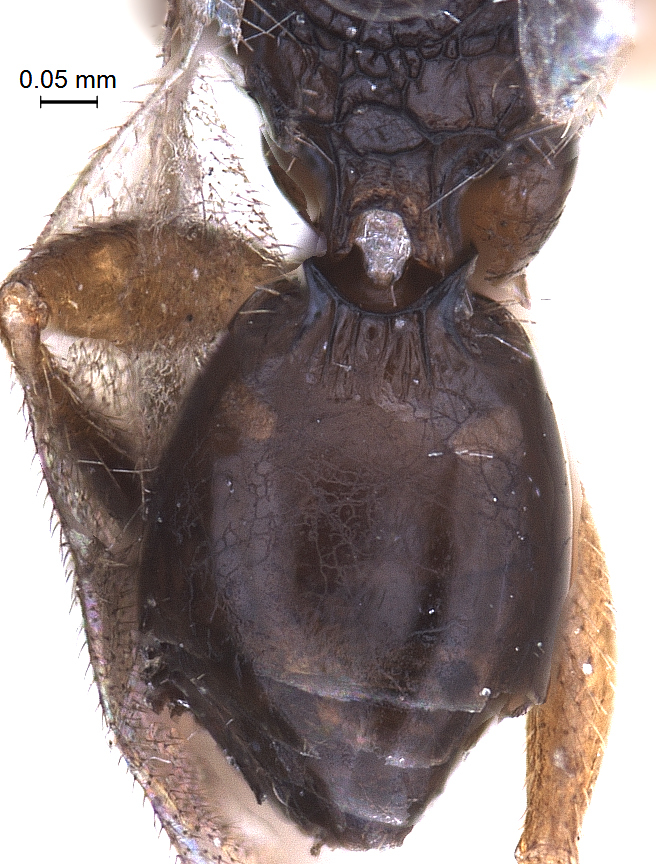
Male, metasoma dorsal view.

**Metasoma:** (Fig. 10); (length/width/height: 484/390/340); metasoma smooth; gastral collar well developed and widened more than that of propodeal strip, with syntergal translucent patch. Four strong transverse gastral carinae present on basal portion of metasoma. A pair of paler gastrocoeli seen quite below gastral collar; largest tergite, T3 occupying 72% of metasomal length. Metasoma held in an elevated manner, above propodeal plane (Fig. 1) (best visible in lateral view). Genitalia with short basal ring, volsella widely separated basaly with long terminal setae on each parossiculi (Fig. 15) (harpe damaged).

##### Female (Paratype)

**Figure 11.**
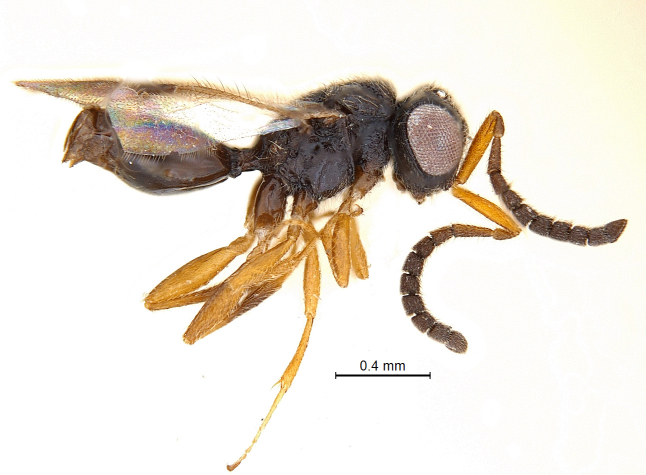
Female habitus.

**Figure 12.**
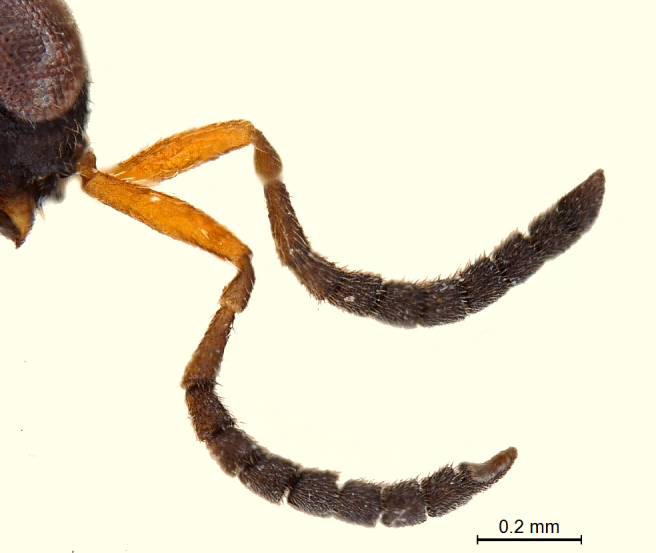
Female antenna.

**Figure 13.**
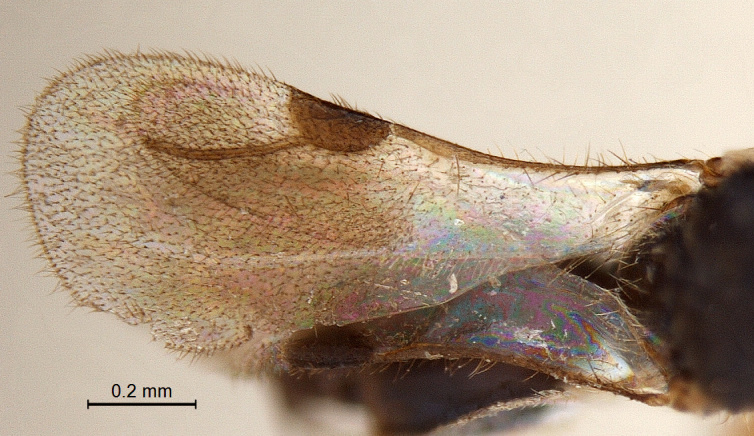
Female forewing.

**Figure 14.**
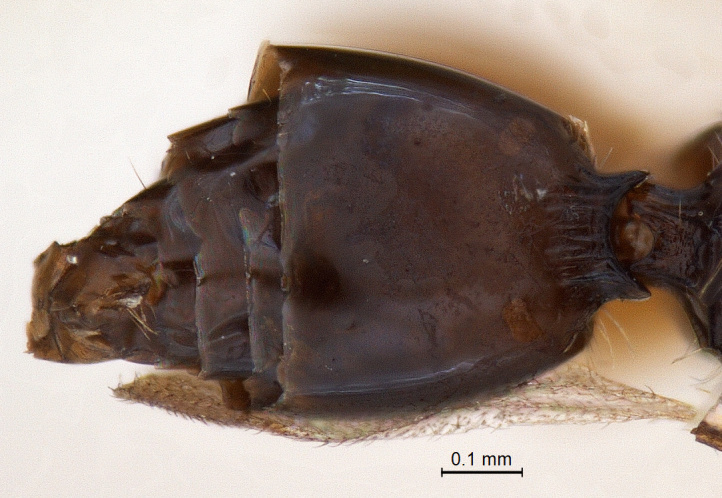
Female, metasoma dorsal view.

**Figure 15.**
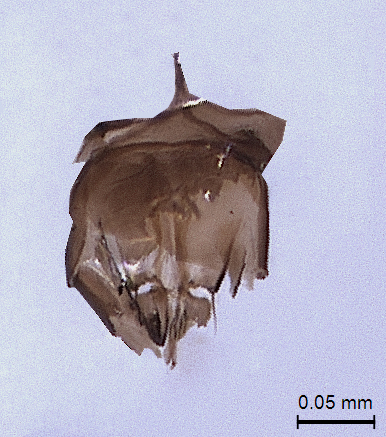
Male genitalia ventral view.

Figs 11, 12, 13, 14.

Body length 1.68 mm. Body colouration and the morphological features of head, mesosoma and metasoma are the same as holotype, except the measurements of antennal segments, proportion of radius and pterostigma and length of metasoma.

Scape more than 4 × as long as wide and A3 more than 3 × as long as wide. Pedicel slender to succeeding antennal segments. Length/width measurements of antennal segments: scape (321/73), pedicel (103/51), A3 (170/51), A4 (97/65), A5 (86/79), A6 (83/90), A7 (90/87), A8 (79/84), A9 (80/85), A10 (87/78), A11 (124/68).

DFIm 52% of head width and fore wing infuscation much darker compared to paler infuscation in male fore wing. T3 occupying 57% of metasomal length.

#### Diagnosis

*Conostigmus neotubifer* sp. n. can be diagnosed by the following features.

Head transverse, wider than mesosoma. Male antenna subequal to body length with A3 highly slender, 4.8 × as long as wide and subequal to scape. Flagellar segments in male quasi cylindrical with very slight serrations basally. Female antenna with scape more than 4 × as long as wide and A3 more than 3 × as long as wide. Ocellar triangle isosceles, narrow based, short and raised in both sexes. Facial sulcus extending to intertorular carina in both sexes. Preoccipital furrow distinct. Supraclypeal furrow absent. DFIm 0.57% of head width in male and 0.52% of head width in female. Eyes densely pubescent. Dorsal margin of propodeal foramen ‘U’ shaped in dorsal view; median propodeal projection absent. Sternaulus absent. Metasoma dorsally elevated from the propodeal axis. Forewing infuscus, darker in female and paler in male.

#### Biology

Unknown.

#### Etymology

The species is named '*neotubifer*', since this species resembles *Conostigmus tubifer* ('*Neo*' in Latin = 'new').

#### Distribution

INDIA, Andaman Nicobar island, Port Blair.

## Discussion

The proposed new species is placed under *Conostigmus*, since its putative sister *Conostigmus tubifer* belongs also to *Conostigmus* and can be justified mainly by the presence of independent parossiculi in male genitalia, one of the diagnostic characters for the subgenus *Conostigmus* s.str. in Dessart and Cancemi (1989). *Conostigmus tubifer* is unique among the Oriental species because of its affinity to *Dendrocerus* due to the quasi cylindrical appearance of male antennal segments, broad based nature of ocellar triangle (Dessart 1997), along with characters as mentioned (Table 1). Except in the ocellar ratio *Conostigmus neotubifer* resembles *Conostigmus tubifer*, in aspects regarding head, mesosoma and metasoma (Table 1) and also in having an upwardly elevated posture of metasoma. This intermixing of character states of the two genera *Conostigmus* and *Dendrocerus* in *Conostigmus tubifer* and *Conostigmus neotubifer* sp. n. emphasizes the uncertain state of the current classification of Ceraphronoidea.

In *Conostigmus neotubifer* sp. n., OOL > POL (ocellar triangle is narrow based), while in *Conostigmus tubifer* OOL < POL (ocellar triangle is broad based). It needs special mention that the illustration No. 94, in page 87 and the description of *Conostigmus tubifer* in Dessart (1997), regarding ocelli, is a mismatch. In the description of the holotype male, Dessart clearly states, supported by numerical values, that the ocellar triangle is broad based, which is in contradiction to the medial placement of ocellar triangle in the illustration. As the holotype of *Conostigmus tubifer* could not be traced, we had no means than to go by the descriptive part of the original literature, since it is supported by numerical values. Though NHM is mentioned as the type depository of *Conostigmus tubifer*, in Johnson and Musetti (2004) and Dessart (1997), on enquiry at NHM, we were informed that the types were never deposited there in reality.

In addition, the differences in the following characters also ensure that *Conostigmustubifer* and *Conostigmusneotubifer* are not conspecific.

1. Forewing infuscation extending further to the distal margins beyond pterostigma and radius in *Conostigmus neotubifer* sp. n. (restricted under stigma and radius in *Conostigmus tubifer*).
2. Facial groove distinct in both sexes in *Conostigmus neotubifer* sp. n. (in *Conostigmus tubifer* facial groove present in male and absent in female).
3. Pterostigma is 2.4 × longer than wide in *Conostigmus neotubifer* sp. n. (only 2 × longer than wide in *Conostigmus tubifer*).
4. DFIm value is 52% in female in *Conostigmus neotubifer* sp. n. (46% in female in *Conostigmus tubifer*).
5. Scape and A3 of male subequal in length in *Conostigmus neotubifer* sp. n. (in *Conostigmus tubifer*, A3 is shorter (only 0.85 ×) than scape).
6. In *Conostigmus neotubifer* sp. n., A3 of female antenna is 3 × longer than wide and scape 2 × longer than A3 (in *Conostigmus tubifer*, A3 is only two 2 × longer than wide and scape is 4 × longer than A3).

## Supplementary Material

XML Treatment for Conostigmus neotubifer

## Figures and Tables

**Table 1.** Characters for distinguishing *Conostigmus* and *Dendrocerus*.

Characters	* Conostigmus *	* Dendrocerus *	*Conostigmus neotubifer* sp. n.	*Conostigmus tubifer*
**Ocellar triangle**	Usually with narrow base	Usually with broad base	* Conostigmus *	* Dendrocerus *
**Shape of head**	Usually globular	Usually transverse	* Dendrocerus *	* Dendrocerus *
**Intertorular carina**	Complete and with a median projection	Complete or incomplete but without a median projection	* Conostigmus *	* Conostigmus *
**Notauli**	Smoothly curved towards anterior angles of mesoscutum	Sharply angulate towards anterior angles of mesoscutum	* Conostigmus *	* Conostigmus *
**Mesoscutum**	Anteriorly narrowed	Quadrate	* Dendrocerus *	* Dendrocerus *
**Basal antennal segments of male**	Cylindrical	Serrate, triangular, ramose or quasi cylindrical	Quasi cylindrical as in *Dendrocerus*	Quasi cylindrical as in *Dendrocerus*
**Shape of scutellum**	Flat	Convex or raised	* Conostigmus *	* Conostigmus *
**Parossiculi**	Independent	Fused	* Conostigmus *	* Conostigmus *
